# Resuscitation with Blood Products Attenuates Endothelial Glycocalyx Shedding but not the Acute Inflammatory Response to Injury in a Military-relevant Preclinical Porcine Model of Traumatic Hemorrhagic Shock

**DOI:** 10.1097/SHK.0000000000002740

**Published:** 2025-10-16

**Authors:** Robert Purcell, Jessica Katy Skelton, Alexander Stoll, Dominic Jenner, Sarah Ann Watts, Emrys Kirkman

**Affiliations:** CBR Division, Defence Science and Technology Laboratory, Porton Down, Salisbury, UK

**Keywords:** Fresh-frozen plasma, fresh whole blood, hemorrhage, inflammation, packed red blood cells, prolonged care, trauma

## Abstract

Traumatic injury induces a complex host response, comprising endothelial damage, and simultaneous pro- and anti-inflammatory responses. These may contribute to complications seen in some patients days or weeks later. Although there is ever-increasing evidence showing that resuscitation with blood products improves survival, their impact on the host response remains unclear. A terminally anesthetized Large White pig model of traumatic hemorrhagic shock (THS) and prolonged care evaluated different resuscitation fluids (saline, fresh-frozen plasma, packed red blood cells and fresh-frozen plasma [1:1], or fresh whole blood [n = 9 per group]). Serial blood samples were collected for enzyme-linked immunosorbent assay, hematology, and flow cytometry, and *postmortem* tissue samples collected for RT-qPCR and immunohistochemistry. THS significantly increased circulating markers of endothelial activation (angiopoietin-2 and von Willibrand factor antigen; both *P* < 0.001) and glycocalyx shedding (hyaluronic acid; *P* < 0.001). THS also elicited a robust inflammatory response, with significant elevations in circulating interleukin-6 and high mobility group box 1 (both: *P* < 0.001), neutrophilia (*P* < 0.001), lymphopenia (*P* < 0.001), and increased inflammatory gene expression across a number of tissues. Compared with saline, resuscitation with blood products reduced hyaluronic acid (*P* < 0.001) but not angiopoeitin-2 or von Willebrand factor antigen (both: *P* > 0.05). The effect of blood products on peripheral cytokine concentrations or immune cell populations was minimal, nor did they significantly alter tissue inflammatory gene expression, neutrophil, or lymphocyte number compared with saline-treated animals. These data suggest resuscitation with blood products can protect the endothelial glycocalyx, but they have little impact on the acute (<8 hours) host response(s) to THS and prolonged care compared to animals treated with saline.

## Graphical abstract

**Figure:**
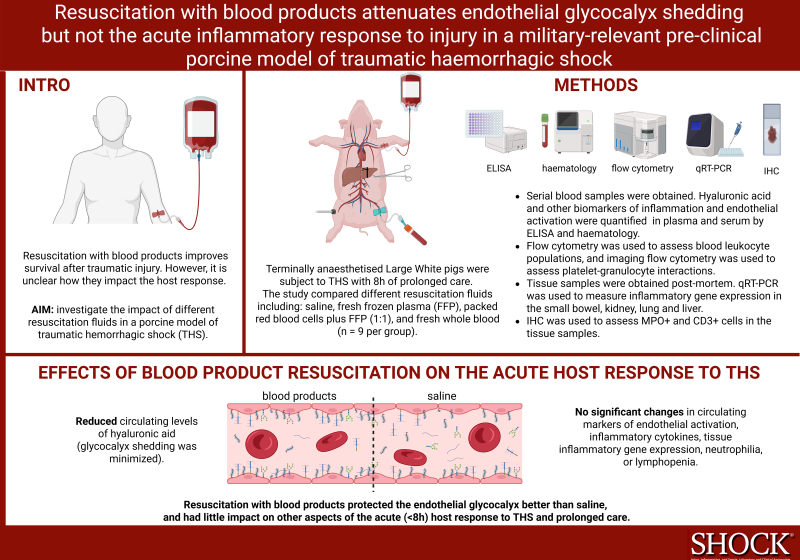

## INTRODUCTION

Traumatic injury is one of the leading causes of death worldwide and is particularly relevant in military settings. Although improvements in treating and managing hemorrhage in critically injured patients have reduced mortality, a number of patients will develop infections and/or multiple organ dysfunction syndrome (MODS) in the subsequent days and weeks (1–3). These complications are associated with a worse prognosis and further burden healthcare systems because of prolonged hospitalization (1,2,4). Although the pathophysiological processes are not completely understood, disturbances to the endothelium and the immune response are believed to be heavily involved (5,6). Traumatic injury initiates a complex and dynamic host response in the minutes after the initial insult, characterized by concurrent pro- and anti-inflammatory responses (7). These are largely driven by the release of damage-associated molecular patterns (DAMPs), such as high mobility group box 1 (HMGB-1), from damaged cells after tissue injury (7). DAMPs activate immune cells and the endothelium, stimulating the release of cytokines and chemokines that initiate and propagate the systemic inflammatory response (6,7). This inflammatory environment, coupled with other manifestations of traumatic injury such as catecholamine release, damages the vascular endothelium. This so-called “endotheliopathy of trauma” occurs within minutes of the initial insult (8) and is characterized by endothelial glycocalyx shedding and endothelial activation (9). As a result, the integrity of the endothelial barrier is disrupted, resulting in edema, inflammation, and, potentially, organ damage (10). Biomarkers of endotheliopathy, such as syndecan-1 (11), hyaluronic acid (HA) (12), and angiopoietin-2 (Ang-2) (13), are elevated after injury, and correlate with injury severity (13,14), MODS (8), and increased mortality (11,14). The systemic inflammatory response also initiates changes in circulating leukocyte populations, with temporal changes in number, activation status, and function occurring in the hours that follow injury (15–19).

Limiting the volume of crystalloid and prioritizing resuscitation with blood products is a key principle of damage control resuscitation (20). Although some reports show no benefit (21), increasing evidence suggests that prehospital resuscitation with blood products improves survival (22,23). It is thought that blood products may exert their benefits by increasing tissue perfusion, limiting or repairing damage to the endothelium, and correcting coagulopathy (20). It has been shown that the volume of crystalloid administered is associated with MODS (3), suggesting that early intervention with blood products may improve outcomes in these patients. That said, very little is known about how early resuscitation with blood products influences immune response(s) and subsequent tissue damage, especially in the context of prolonged casualty care. We have demonstrated in our model of traumatic hemorrhagic shock (THS) that resuscitation with blood/blood components, initiated in response to hypotension, improves pathophysiological outcomes compared with saline (24). We postulated that blood/blood components limit and/or treat the oxygen debt, and consequently reduce endothelial dysfunction and inflammation. Therefore, the aim of this study was to explore the cellular and molecular responses that occur in our military-relevant porcine model of THS and examine whether the type of resuscitation fluid affects these responses.

## MATERIALS AND METHODS

This was a randomized study in terminally anesthetized female cross-bred Large White pigs (~50 kg); animals were purchased from a UK commercial supplier. This model of THS, combining tissue injury and hemorrhage, was developed to recapitulate prolonged evacuation times that are relevant to military settings, with an extended prehospital resuscitation phase. This study underwent local ethical review and was carried out under the authority of Animals (Scientific Procedures) Act 1986. The study presented here utilizes samples obtained from a previously published study, where the full detailed experimental protocol has been described (24); a brief description is given later.

### Porcine model of THS

**Fig. 1.**
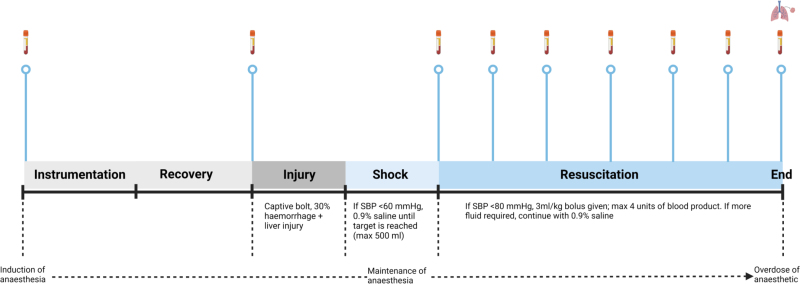
**Porcine model of traumatic hemorrhagic shock.** Experimental timeline highlighting the various phases of the experimental protocol, including blood sampling times (minutes) and tissue collection. SBP, systolic blood pressure. This figure was created with BioRender.com.

A timeline of the experimental protocol is shown in Figure 1. Intramuscular midazolam hydrochloride (Hypnovel, Roche Products Ltd, Welwyn Garden City, UK) (0.1 mg/kg) was administered 15 minutes before induction of anesthesia with isoflurane (Isoflurane-Vet, Meriel Animal Health Ltd, Bracknell, UK). Surgical anesthesia was maintained with isoflurane (1% - 2%) in oxygen and nitrous oxide (40:60) using a Penlon AV-S ventilator (Penlon Ltd, Abingdon, UK). Animals were instrumented for continuous physiological monitoring, and a midline laparotomy was performed to cannulate the bladder, perform a splenectomy, and place a snare around the left medial lobe of the liver. Upon completion of instrumentation, surgical anesthesia was converted from isoflurane to intravenous alphaxalone (Alfaxan 10 mg/mL single use, Jurox (UK) Ltd, Malvern Link, UK), and nitrous oxide was discontinued. After a suitable washout period on enriched air, animals were weaned off the ventilator. Animals were allowed to breathe spontaneously for the remainder of the experiment, unless during the injury and resuscitation phases they displayed marked respiratory depression (respiratory rate <10 breaths per minute). At which point pressure-controlled synchronized intermittent mandatory ventilation was initiated (Drager Evita Infinity V500, Draeger Medical UK Ltd, UK). Animals’ physiology was then allowed to recover for 60 minutes before baseline measurements and samples were taken.

The injury phase comprised three components, first a soft tissue injury (four shots to the right thigh) was created using a captive bolt (Cash Special, Accles and Shelvoke, Sutton Coldfield, UK), after a controlled hemorrhage (30% blood volume) at an exponentially reducing rate, and lastly the snare around the liver lobe was pulled to initiate an uncontrolled hemorrhage. Animals then entered the shock phase, which lasted 30 minutes. After the shock phase, animals then entered the resuscitation phase, where the treatment groups diverged based on the fluid they received. Here, animals were randomized to receive either 0.9% saline (Aqupharm, York, UK), fresh-frozen plasma (FFP), packed red blood cells (PRBC), and FFP (1:1 ratio) or fresh whole blood (FWB); n = 9 animals per group. An additional group of animals received no treatment during the resuscitation phase (n = 9), but survival was too short to take serial blood samples, and so this group is not included in the results presented here. Blood products were collected and prepared from donor animals as previously described (25) in accordance with UK donation guidelines. Before use, all donor products were forward and reverse matched to the recipient’s blood. During the resuscitation phase, a 3 mL/kg bolus of fluid was administered when systolic blood pressure fell below 80 mmHg; with a maximal number of 4 units of the respective blood product given to any one animal. At the end of the experiment (480 minutes after the start of resuscitation), or when the SBP <10 mmHg, animals were culled with an overdose of pentobarbitone (Euthatal, Merial Animal Health Ltd, Harlow, UK). Body temperature was maintained at ~38°C throughout the experiment using external heating/cooling and blankets as appropriate.

### Blood and tissue sampling

Arterial blood was serially sampled at defined time points throughout the experiment (Fig. 1) for flow cytometry, hematology, platelet function, and biomarker analysis. For flow cytometric analysis, blood was collected into citrated vacutainers (BD Biosciences, New Jersey) and processed as described later. For plasma, blood was collected into ethylenediaminetetraacetic acid (EDTA) and sodium citrate vacutainers (BD Biosciences), centrifuged (1300 × *g* for 10 minutes at room temperature), and the plasma collected. For serum, blood was collected into Rapid Serum Tubes (BD Biosciences) and allowed to clot for at least 10 minutes before centrifugation (1500 × *g* for 10 minutes at room temperature) to separate the serum. Plasma and serum were frozen at −80°C until analysis. To assess platelet function, blood was collected into hirudin vacutainers (BD Biosciences) and analyzed as described later. For hematology, blood was collected into EDTA vacutainers and analyzed by a laboratory analyzer within 24 hours of sample collection (Advia 2120i, Siemens Healthcare GmbH, Germany).

*Postmortem* samples were collected from liver, lung, kidney, and small bowel (jejunum) for immunohistochemistry and gene expression analysis. For immunohistochemistry, samples were collected into 10% neutral buffered formalin (Sigma-Aldrich, Burlington, Vermont) by immersion fixation and processed as described later. For gene expression, representative sections (~3 mm^3^) of tissue were placed into RNAlater (ThermoFisher Scientific, Waltham, Massachusetts), refrigerated overnight, before being stored at −80°C.

To act as a control tissue for immunohistochemistry and gene expression analysis, a naïve control group (n = 9) was included in the study using samples from our naïve tissue Bio-bank. Tissues from this Bio-bank were collected in the same manner as described earlier, from Schedule 1 culled (overdose of pentobarbitone) age, weight, and sex-matched Large White pigs.

### Platelet function

Platelet function in hirudinized whole blood was assessed using ADPtest and ASPItest on a Multiplate analyzer (Roche, Basel, Switzerland), according to the manufacturer’s instructions.

### Biomarker analysis

Plasma and serum biomarkers were quantified by enzyme-linked immunosorbent assay (ELISA) according to the manufacturer’s instructions. Standards and samples were run in duplicate, and samples were diluted where appropriate. HA and Ang-2 were measured in serum (both R&D Systems, Minneapolis, Minnesota), while HMGB-1 (Tecan, Zurich, Switzerland), tumor necrosis factor α (TNFα; R&D Systems), and interleukin-6 (IL-6; R&D Systems) were quantified in EDTA plasma. von Willebrand factor (vWF) antigen was assessed in citrated plasma using a commercially available assay on an ACL TOP 300 CTS analyzer (Werfen, Warrington, UK).

### Flow cytometry and imaging flow cytometry

Flow cytometry was used to assess leukocyte phenotype, while imaging flow cytometry was used to assess platelet–granulocyte interactions; the methods are detailed in full in Supplementary Text, https://links.lww.com/SHK/C650, and antibody panels are described in Supplementary Tables 1 and 2, https://links.lww.com/SHK/C650.

### Gene expression

RNA was extracted from tissue samples that had been stored in RNAlater (ThermoFisher Scientific). For this, 30 mg of tissue was homogenized in RLT lysis buffer using a rotor-stator homogenizer (Tissue Ruptor II; Qiagen, Hilden, Germany) and total RNA extracted using a commercially available kit (RNeasy; Qiagen). The quantity and quality of the extracted RNA were measured using a spectrophotometer (Nanodrop one, ThermoFisher Scientific). After this, 500 ng RNA was reverse-transcribed to cDNA using a commercially available kit (RT^2^ First Strand Kit; Qiagen) and stored at −20°C until analysis. Tissue gene expression was analyzed using custom RT^2^ Profiler PCR arrays according to the manufacturer’s instructions (Qiagen) using a QuantStudio 7 Flex Real-Time PCR System (ThermoFisher Scientific). Details of the final RT^2^ Profiler PCR arrays can be found in Supplementary Table 3, https://links.lww.com/SHK/C650. All samples were run in duplicate, none of which showed signs of genomic DNA contamination (*Ct* > 35). Relative gene expression compared with the naïve control group was calculated using the ΔΔ*Ct* method. For this, *Ct* values of the target genes were normalized to the geometric mean *Ct* value of three housekeeping genes, before being compared with the naïve control group. Data are presented as fold change with respect to the naïve control group.

### Immunohistochemistry

Samples fixed in 10% neutral buffered formalin were processed and embedded in paraffin wax, and 4-µM tissue sections were cut using a rotary microtome (Shandon Finesse ME; Epredia, Cheshire, UK) onto charged slides before immunolabelling for myeloperoxidase (MPO) and CD3. The method is detailed in full in Supplementary Text, https://links.lww.com/SHK/C650, and details of primary and secondary antibodies, conditions, and antigen retrieval can be found in Supplementary Table 4, https://links.lww.com/SHK/C650. Slides were digitally scanned (Olympus Slideview VS200-BU; Tokyo, Japan) and the number of MPO^+^ and CD3^+^ cells quantified using QuPath (version 0.4.3). MPO^+^ and CD3^+^ cells are expressed as a percentage of the total cell count.

### Statistical analysis

All data were assessed for normality and subjected to transformation if necessary. Continuous variables with repeated measures over time were analyzed by linear mixed model analysis of variance (ANOVA), using preinjury baseline values as the covariate for the injury/shock phase analysis, and the end-shock value as the covariate for the resuscitation phase. Unless indicated otherwise, analysis refers to the main effect of time, treatment, and treatment by time interaction. Where a main treatment effect was seen, planned comparisons were made between treatment groups as indicated. The one exception in this respect was HMGB-1, which was resistant to transformation and where the effects of shock and resuscitation were determined using a Wilcoxon signed-rank test over Groups to establish a Time effect, interpreted as a Shock Effect or Resuscitation Effect according to the appropriate dataset. Gene expression data were subjected to log-transformation followed by a Bartlett’s test of homogeneity of variance to determine, on a variable by variable basis, whether subsequent group analysis should be performed using a one-way ANOVA of groups of Unequal Variance followed by Welch two-sample *t* test, or a Dunnett’s ANOVA (comparison of each treatment group to naïve controls) or Tukey ANOVA (comparison of each treatment group except naïve). *P* < 0.05 was taken as statistically significant. All analyses were performed using the R Statistics Package (R v4.1.3, R Studio, Boston, Massachusetts). All data are presented as mean ± SEM unless indicated otherwise.

For graphs presented within this article, the *x* axis times refer to the following (also shown in Fig. 1): −120 minutes: presurgery sample; −60 minutes: baseline sample (taken just before the injury); 0 minutes: the end of shock phase (sample taken after the injury and 30 minute shock phase and just before the start of resuscitation); 45, 90, 180, 270, 360, and 450 minutes: samples collected throughout the resuscitation phase.

## RESULTS

The primary outcomes from the study, including survival time, physiological parameters, and resuscitation fluid volume, have been previously published (24). To briefly summarize, all animals that received FWB, FFP, and PRBC:FFP, and 7/9 animals that received saline survived until the end of the resuscitation phase. In contrast, none of the animals in the no-treatment group survived to the end (7/9 died within 90 minutes), demonstrating the severity of the model. Animals resuscitated with saline exhibited a more variable and severe physiological response (e.g., degree of shock (24), and severity of tissue injury [manuscript in preparation]). These animals required more fluid than those given blood products (24). Due to the short survival times, data from the no-treatment group are not included in the results presented here. Unless otherwise stated, no differences after surgery were observed in circulating biomarker or cell concentrations.

### Circulating markers of inflammation

**Fig. 2.**
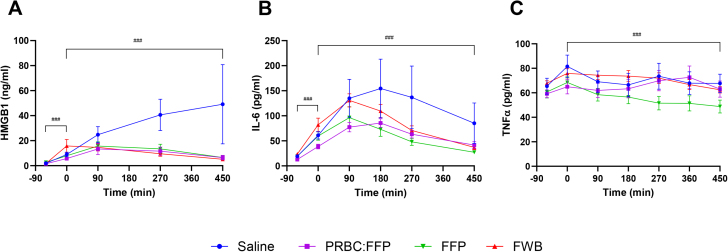
**Tissue injury and hemorrhagic shock increase circulating markers of inflammation.** Circulating levels of (A) HMGB-1, (B) IL-6, and (C) TNFα were quantified by ELISA. Data are presented as mean ± SEM (n = 9 per group). Main effect of time: ###*P* < 0.001. ELISA, enzyme-linked immunosorbent assay; FFP. Fresh-frozen plasma; FWB, fresh whole blood; HMGB-1, high mobility group box 1; IL-6, interleukin-6; PRBC:FFP, packed red blood cells:fresh-frozen plasma; TNFα, tumor necrosis factor α.

For analytes in Figure 2, presurgery (−120 minute) levels are not presented due to the levels being at, or below, the limit of detection for their respective assays (data not shown). At the end of the shock phase, HMGB-1 (Fig. 2A) and IL-6 (Fig. 2B) concentrations were significantly elevated compared with baseline values (both: *P* < 0.001); however, TNFα (Fig. 2C) levels remained unchanged (*P* = 0.133). During the resuscitation phase, all three analytes changed significantly with time (*P* < 0.001). In the saline group, HMGB-1 levels continued to rise, whereas after resuscitation with blood/blood products, the levels fell toward baseline levels; however, this difference did not attain statistical significance (*P* = 0.054). IL-6 concentrations initially increased (peaking 90–180 minutes after the start of resuscitation) before gradually declining until the end of the experiment. A difference between groups (*P* = 0.015) and a different pattern between groups (*P* < 0.001) was observed. Here, FFP was significantly different from saline (*P* = 0.007) and PRBC:FFP (*P* = 0.032); there were no other differences between groups (all: *P* > 0.05).

### Endotheliopathy

**Fig. 3.**
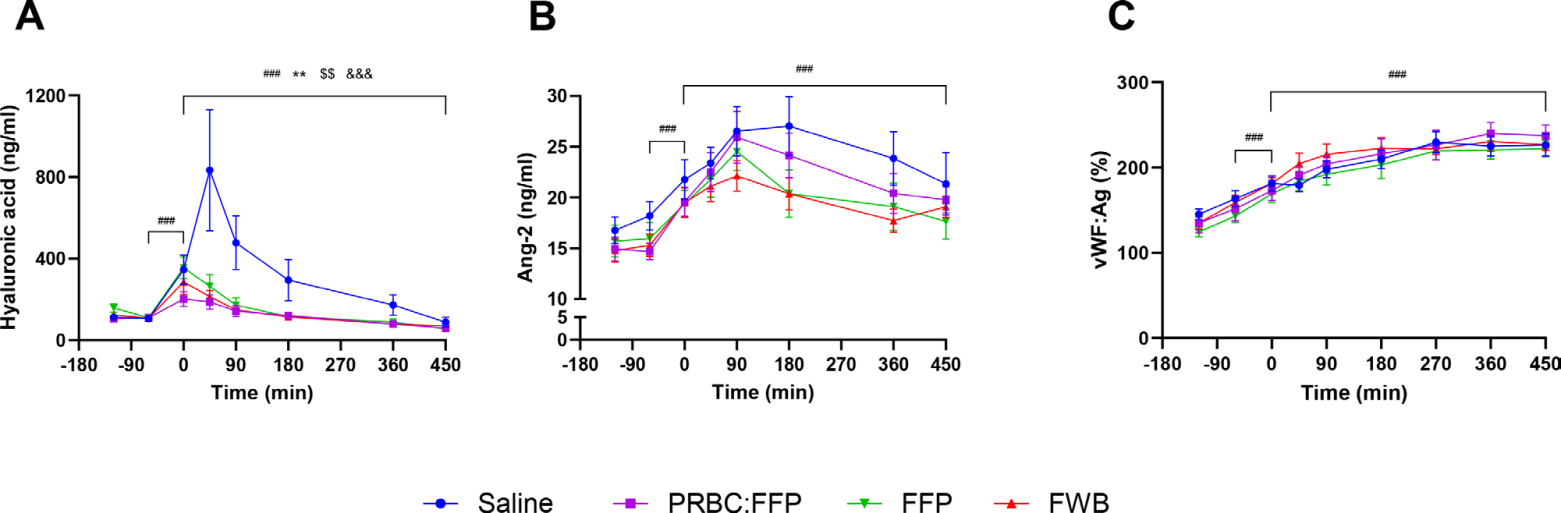
**Tissue injury and hemorrhagic shock induce endotheliopathy.** Circulating levels of (A) hyaluronic acid and (B) Ang-2 were quantified by ELISA, and (C) vWF:Ag was quantified using a commercially available assay on an ACL TOP 300 CTS analyzer. Data are presented as mean ± SEM (n = 9 per group). Main effect of time: ###*P* < 0.001. Difference between treatment groups: FWB *versus* saline: **P < 0.01, PRBC:FFP *versus* saline: $$*P* < 0.01, FFP *versus* saline: &&&*P* < 0.001. Ang-2, angiopoietin-2; ELISA, enzyme-linked immunosorbent assay; FFP, fresh-frozen plasma; FWB, fresh whole blood; PRBC:FFP, packed red blood cells:fresh-frozen plasma; vWF:Ag, von Willebrand factor antigen.

HA was elevated at the end of the shock phase compared with baseline levels (*P* < 0.001) and changed significantly over time during the resuscitation phase (*P* < 0.001). In the resuscitation phase, HA was significantly different between treatment groups (*P* < 0.001), and the pattern of response between groups was also different (*P* = 0.002). (Fig. 3A). While levels of HA continued to rise in the saline group (peaking after 45 minutes), resuscitation with blood products attenuated HA levels. As such, levels of HA in the saline group were significantly higher than those seen in FWB (*P* = 0.001), PRBC:FFP (*P* = 0.008), and FFP (*P* < 0.001) groups. Ang-2 (Fig. 3B) and vWF antigen (Fig. 3C) were both elevated at the end of the shock phase compared with baseline (both: *P* < 0.001) and changed significantly with time during the resuscitation phase (*P* < 0.001). Concentrations of both analytes increased during the resuscitation phase; however, there were no differences observed between treatment groups (Ang-2: *P* = 0.269, vWF antigen: *P* = 0.436).

### Immune cell populations

**Fig. 4.**
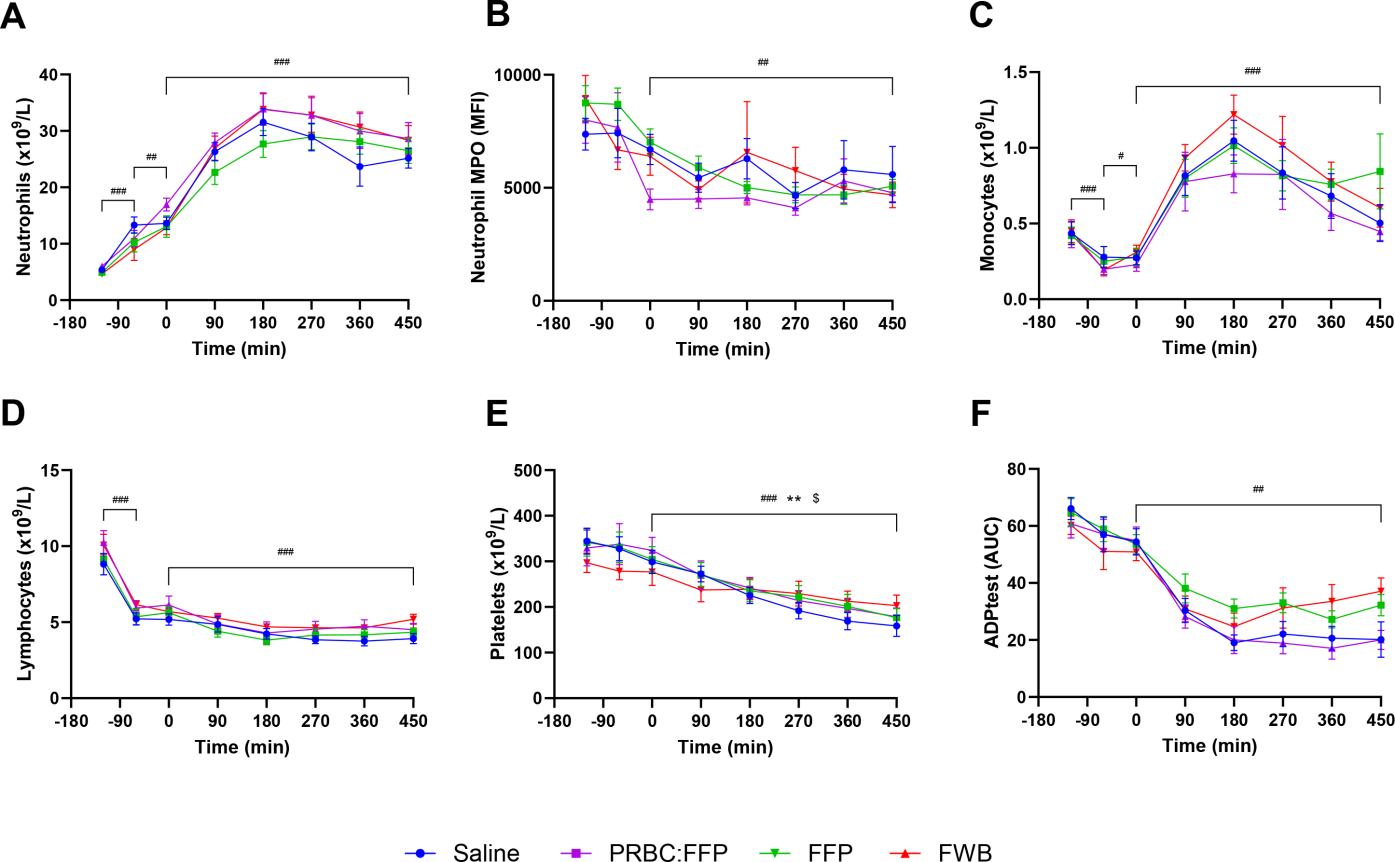
**Tissue injury and hemorrhagic shock alter circulating immune cell populations.** The number of circulating (A) neutrophils, (C) monocytes, (D) lymphocytes, and (E) platelets was quantified using an Advia 2120i hematology analyzer. B, Neutrophil MPO was quantified by flow cytometry, and (F) platelet function in response to ADPtest was assessed by Multiplate. Data are presented as mean ± SEM (n = 9 per group). Main effect of time: #*P* < 0.05, ##*P* < 0.01, ###*P* < 0.001. Difference between treatment groups: FWB *versus* saline: **P < 0.01, FWB *versus* PRBC:FFP: $*P* < 0.05. FFP, fresh-frozen plasma; FWB, fresh whole blood; MFI, median fluorescence intensity; MPO, myeloperoxidase; PRBC:FFP, packed red blood cells:fresh-frozen plasma.

The number of circulating neutrophils (Fig. 4A) was elevated after surgery (*P* < 0.001) and shock (*P* = 0.002) and continued to rise during the resuscitation phase (*P* < 0.001), with no differences between groups (*P* = 0.409). There was also evidence of neutrophil activation, as neutrophil MPO was decreased (indicative of degranulation) throughout the resuscitation phase (*P* = 0.006), although no differences between groups were observed (*P* = 0.806) (Fig. 4B). Surgery reduced the number of circulating monocytes (*P* < 0.001); however, monocyte number was elevated at the end of the shock phase (*P* = 0.011) and in the resuscitation phase (*P* < 0.001); no differences between groups were observed (Fig. 4C). We observed reductions in the proportion of CD80^+^CD86^+^ monocytes during the resuscitation phase (*P* < 0.001), with no differences seen between treatment groups (*P* = 0.294) (Supplementary Figure 2A, https://links.lww.com/SHK/C650).

Lymphocyte number reduced after surgery (*P* < 0.001), and although no changes were seen at the end of the shock phase when compared with baseline (*P* = 0.178), levels declined further in the resuscitation phase (*P* < 0.001); no differences were seen between groups at any stage of the experiment (all *P* > 0.05) (Fig. 4D). The observed lymphopenia may be attributable to changes in T-cell populations as we observed similar findings for CD3^+^ cells (Supplementary Figure 2B, https://links.lww.com/SHK/C650), while changes were also seen basophil and eosinophil number (Supplementary Figure 2, C and D, https://links.lww.com/SHK/C650); further detail is provided in the Supplementary Material, https://links.lww.com/SHK/C650.

Platelet number (Fig. 4E) did not change after the shock phase (*P* = 0.904), but they consistently decreased throughout the resuscitation phase (*P* < 0.001). Platelet number was significantly affected by treatment during the resuscitation phase (*P* = 0.002); the pattern of response between groups was also significantly different (*P* < 0.001). The decline in platelet number was less pronounced in the FWB group compared with the other treatment groups; FWB was significantly different than both saline (*P* = 0.008) and PRBC:FFP (*P* = 0.030) groups, but was not different compared with FFP (*P* = 0.113). During the resuscitation phase, platelet function was impaired in response to both ADPtest (Fig. 4F) and ASPItest (Supplementary Figure 2F, https://links.lww.com/SHK/C650) (both *P* < 0.05). There were no significant differences between groups for ASPItest (*P* > 0.05), but significant differences were observed in response to ADP (*P* < 0.05). Here, platelet function was improved at the latter stages of resuscitation (at both 360 and 450 minutes) by FWB when compared with saline (both: *P* < 0.05) and PRBC:FFP (both: *P* < 0.05). The proportion of granulocytes associated with a platelet was unaffected after the shock phase (*P* = 0.988) but did significantly decline throughout the resuscitation phase (*P* < 0.001); there were no significant differences between groups (Supplementary Figure 2E, https://links.lww.com/SHK/C650).

### Tissue inflammation

**Fig. 5.**
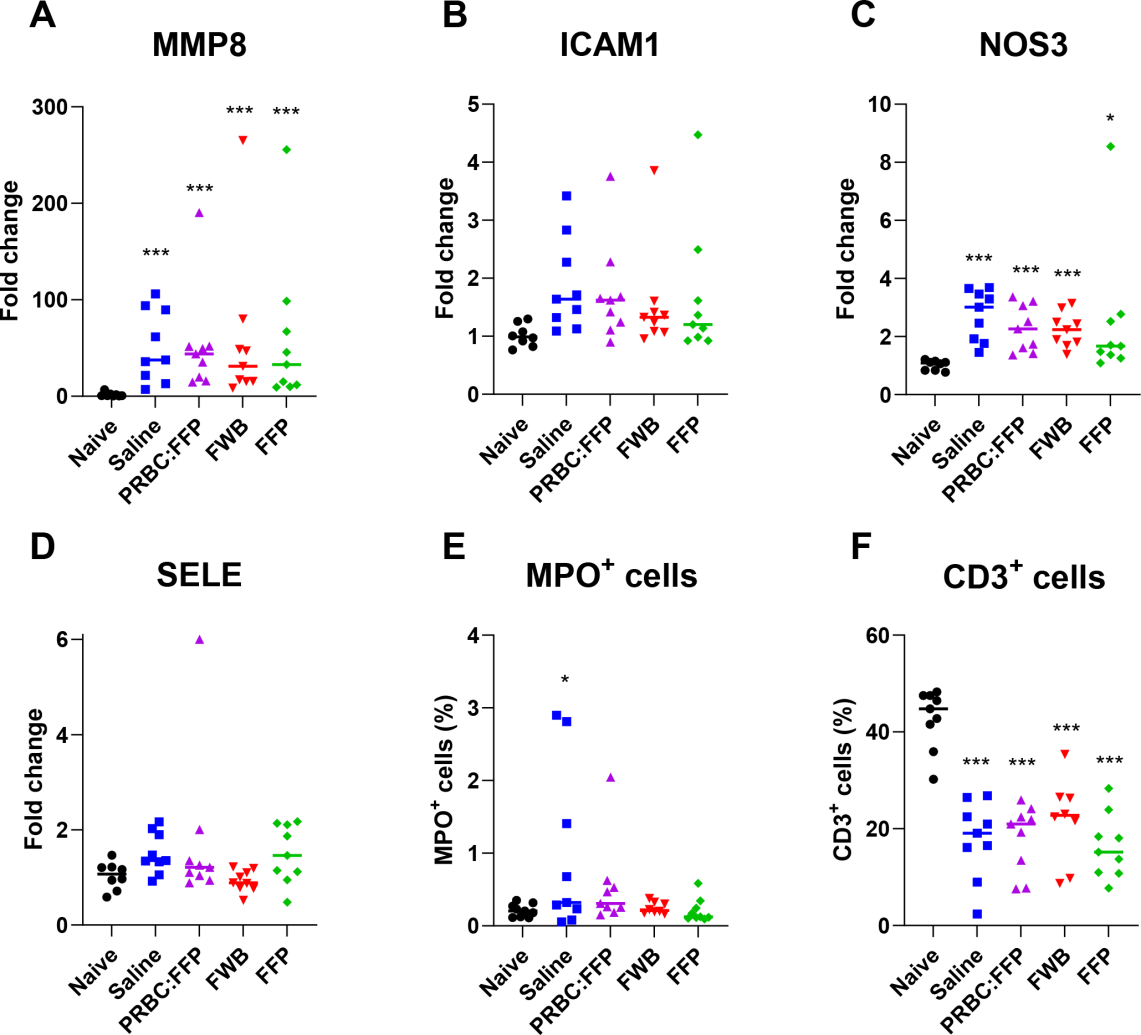
**Gene expression and inflammatory cell number in the small bowel.** Gene expression of (A) MMP-8, (B) ICAM-1, (C) NOS3, and (D) SELE in the small bowel was assessed by RT^2^ profiler PCR arrays; data are presented as fold change compared to the naïve control group. The percentage of (E) MPO^+^ and (F) CD3^+^ cells in the mucosa was quantified by immunohistochemistry. Compared with naïve control group: *P < 0.05, **P < 0.01, ***P < 0.001. Data are presented as median with individual data points (n = 9 per group). FFP, fresh-frozen plasma; FWB: fresh whole blood; ICAM-1: intercellular adhesion molecule-1; MMP-8, matrix metalloprotease-8; MPO, myeloperoxidase; NOS3, nitric oxide synthase 3; PRBC:FFP, packed red blood cells:fresh-frozen plasma; SELE, E-Selectin.

**Fig. 6.**
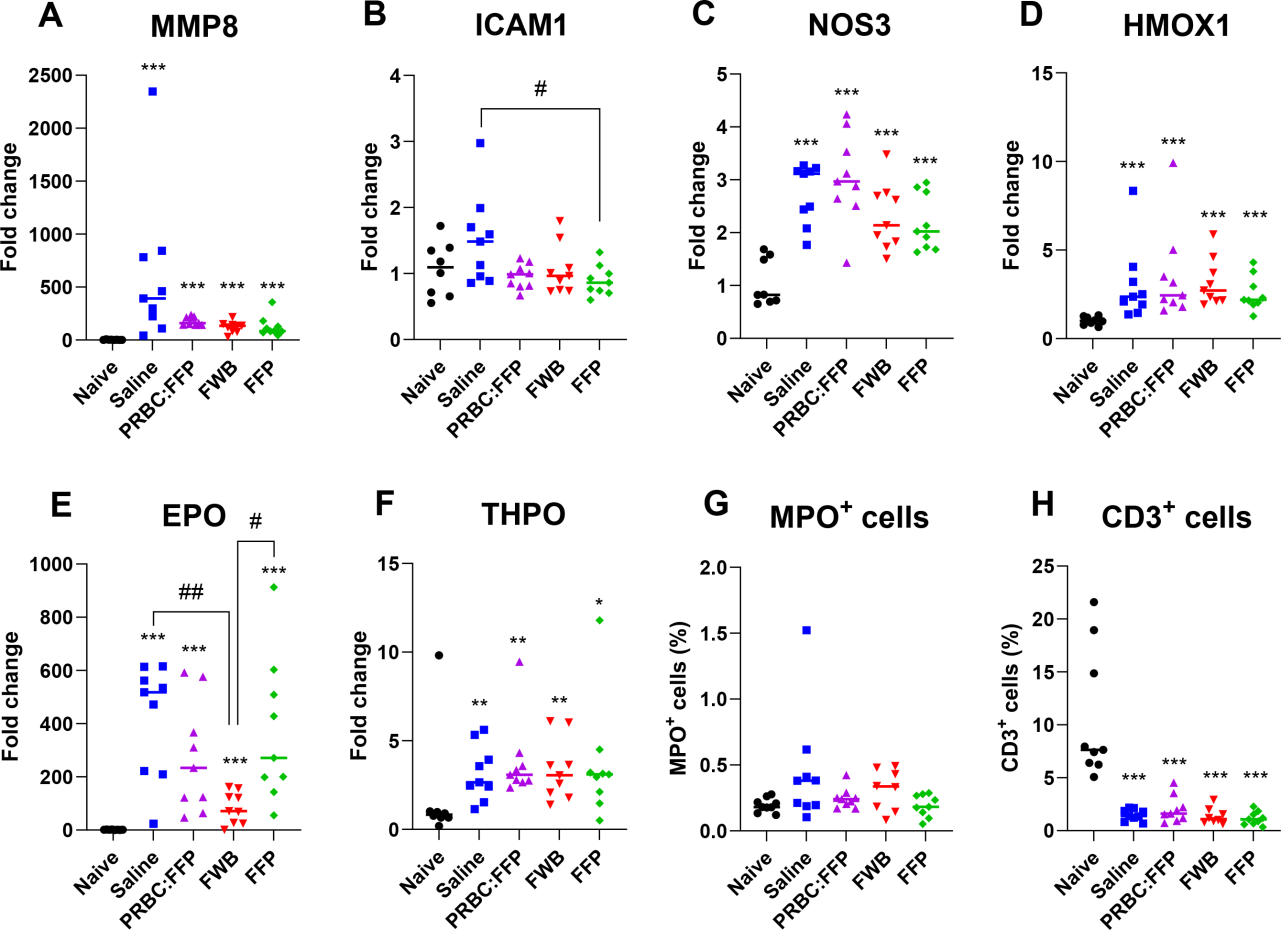
**Gene expression and inflammatory cell number in the kidney.** Gene expression of (A) MMP-8, (B) ICAM-1, (C) NOS3, (D) HMOX1, (E) EPO, and (F) THPO in the kidney was assessed by RT^2^ profiler PCR arrays; data are presented as fold change compared with the naïve control group. The percentage of (G) MPO^+^ and (H) CD3^+^ cells was quantified by immunohistochemistry. Compared with naïve control group: *P < 0.05, **P < 0.01, ***P < 0.001. Treatment comparison: #*P* < 0.05, ##*P* < 0.01. Data are presented as median with individual data points (n = 9 per group). EPO, erythropoietin; FFP, fresh-frozen plasma; FWB, fresh whole blood; HMOX1, heme oxygenase-1; ICAM-1, intercellular adhesion molecule-1; MMP-8, matrix metalloprotease-8; MPO, myeloperoxidase; NOS3, nitric oxide synthase 3; PRBC:FFP, packed red blood cells:fresh-frozen plasma; THPO, thrombopoietin.

**Fig. 7.**
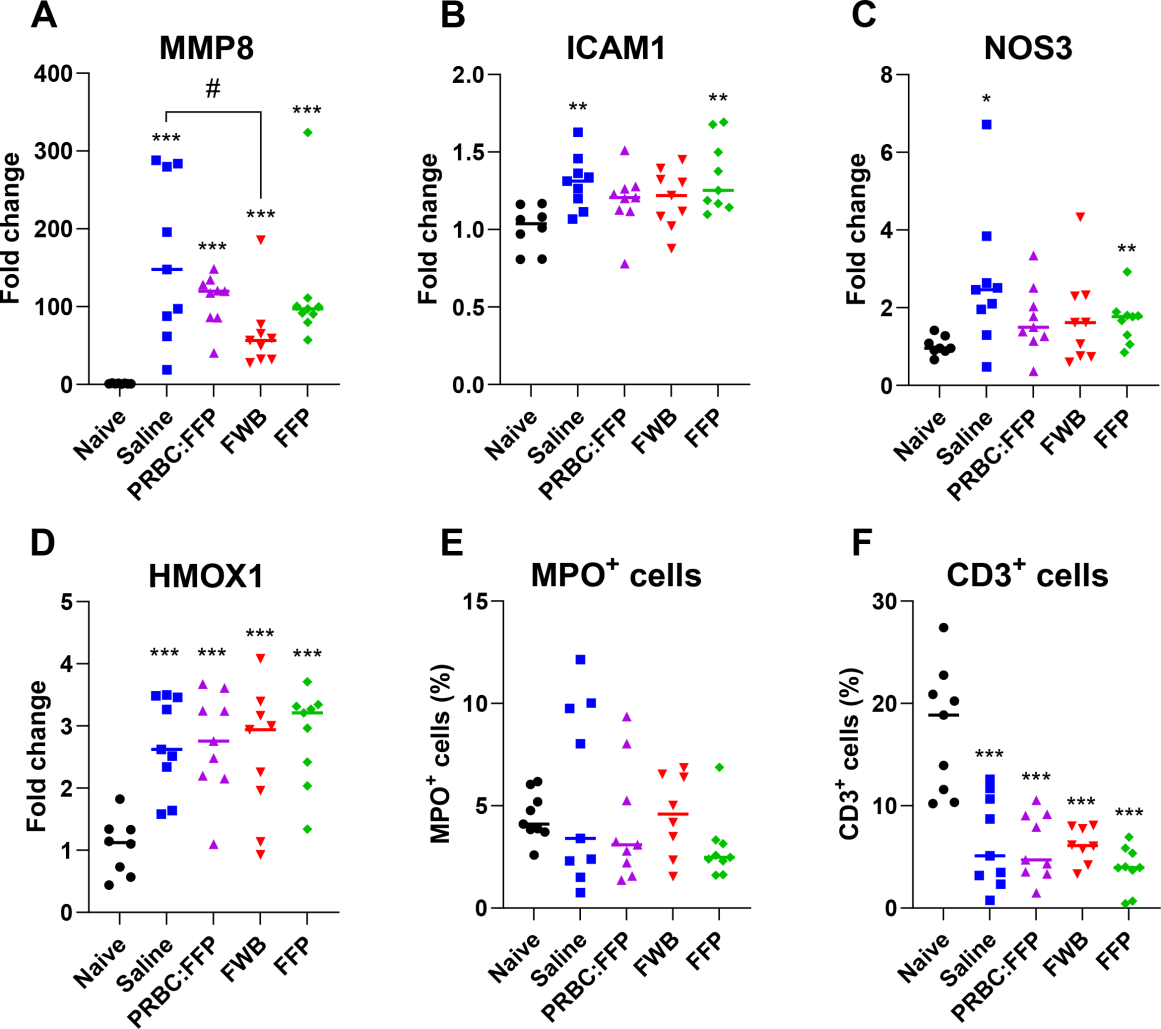
**Gene expression and inflammatory cell number in the lung.** Gene expression of (A) MMP-8, (B) ICAM-1, (C) NOS3, and (D) HMOX1 in the lung was assessed by RT^2^ profiler PCR arrays; data are presented as fold change compared to the naïve control group. The percentage of (E) MPO^+^ and (F) CD3^+^ cells was quantified by immunohistochemistry. Compared with naïve control group: *P < 0.05, **P < 0.01, ***P < 0.001. Treatment comparison: #*P* < 0.05. Data are presented as median with individual data points (n = 9 per group). FFP, fresh-frozen plasma; FWB, fresh whole blood; HMOX1, heme oxygenase-1; ICAM-1, intercellular adhesion molecule-1; MMP-8, matrix metalloprotease-8; MPO, myeloperoxidase; NOS3, nitric oxide synthase 3; PRBC:FFP, packed red blood cells:fresh-frozen plasma.

**Fig. 8.**
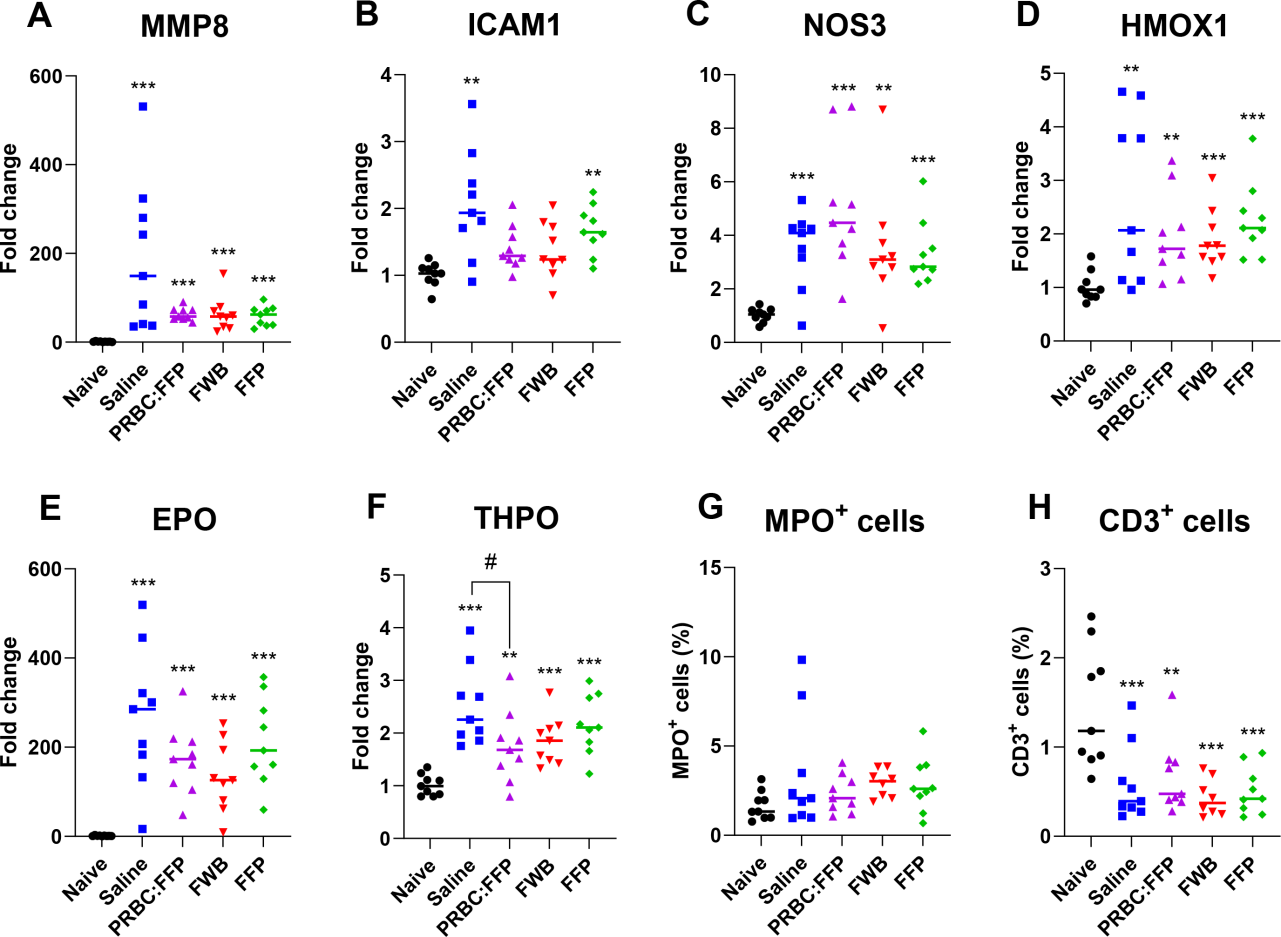
**Gene expression and inflammatory cell number in the liver.** Gene expression of (A) MMP-8, (B) ICAM-1, (C) NOS3, (D) HMOX1, (E) EPO, and (F) THPO in the liver was assessed by RT^2^ profiler PCR arrays; data are presented as fold change compared with the naïve control group. The percentage of (G) MPO^+^ and (H) CD3^+^ cells was quantified by immunohistochemistry. Compared with naïve control group: *P < 0.05, **P < 0.01, ***P < 0.001. Treatment comparison: #*P* < 0.05. Data are presented as median with individual data points (n = 9 per group). EPO, erythropoietin; FFP, fresh-frozen plasma; FWB, fresh whole blood; HMOX1, heme oxygenase-1; ICAM-1, intercellular adhesion molecule-1; MMP-8, matrix metalloprotease-8; MPO, myeloperoxidase; NOS3, nitric oxide synthase 3; PRBC:FFP, packed red blood cells:fresh-frozen plasma; THPO, thrombopoietin.

THS induced significant changes in inflammatory gene expression compared with naïve animals across all tissues studied (Figs 5–8 and Supplementary Figures 3–6, https://links.lww.com/SHK/C650). This included the upregulation of genes associated with inflammation and oxidative stress, such as matrix metalloprotease-8 (MMP-8), intercellular adhesion molecule-1 (ICAM-1), nitric oxide synthase 3 (NOS), and heme oxygenase-1 (HMOX1). However, very few differences were seen between treatment groups. All statistical differences are shown on the respective graphs, and full details of the results are provided in the Supplementary Text, https://links.lww.com/SHK/C650.

In the kidney and liver, all treatment groups had significantly higher levels of erythropoietin (EPO) and thrombopoietin (THPO) gene expression than naïve animals (all: *P* < 0.01). In the kidney, EPO gene expression was significantly lower in the FWB group compared with both the saline- (*P* = 0.007) and FFP- (*P* = 0.012) treated animals. Although there was a trend, EPO expression in the FWB group was not significantly lower than animals that were treated with PRBC:FFP (*P* = 0.06). In the liver, no differences between treatment groups were observed in EPO (*P* = 0.281). There were no differences in THPO expression between treatment groups in either tissue; full details are provided in the Supplementary Text, https://links.lww.com/SHK/C650.

The percentage of MPO^+^ cells in the small bowel (Fig. 5E) was significantly different between groups (*P* = 0.038), with only saline-treated animals having significantly more MPO^+^ cells than the naïve group (*P* = 0.039). Although there was a trend, no significant differences between treatment groups were observed (*P* = 0.078). Across the other tissues studied (Figs 6G, 7E, and 8G), there were no significant differences in the percentage of MPO^+^ cells between groups (all *P* > 0.05). In all tissues (Figs 5F, 6H, 7F, and 8H), the number of tissue-resident T cells was reduced after THS (across all treatment groups) compared with naïve animals (all comparisons: *P* < 0.001 compared with naïve, with the exception of liver PRBC:FFP, where *P* = 0.003). Across all tissues, there were no differences observed between treatment groups (all: *P* > 0.05). Representative images from across the tissues can be found in Supplementary Figure 7, https://links.lww.com/SHK/C650.

## DISCUSSION

In this study, we investigated the host response to hemorrhage and tissue injury in a military-relevant porcine model of trauma and resuscitation. Limiting the volume of crystalloid and prioritizing resuscitation with blood products is a key component of damage control resuscitation (20), but the impact on inflammatory/immune response(s), especially in the context of prolonged prehospital care, is not well understood. In this model, we have previously reported that compared with saline, resuscitation with blood products improves survival, hemodynamics, and shock; with the added advantage of requiring less fluid volume, which is particularly relevant in resource-limited environments, for example, military scenarios (24). In the findings presented here, we demonstrate that our porcine model recapitulates many of the host responses to injury observed in humans. We also show that while resuscitation with blood products can attenuate endothelial glycocalyx shedding, it has little influence on the acute host response to trauma compared with treatment with saline.

In keeping with numerous clinical studies that have reported endothelial glycocalyx shedding (8,9,11,12,14) and endothelial activation (13,26) after trauma, our porcine model induced endotheliopathy, evidenced by elevations in circulating HA, Ang-2, and vWF antigen. These responses may be driven by inflammatory mediators (such as HMGB-1 and IL-6), hypoxia, or locally released enzymes (e.g., MMPs), all of which have been shown to disrupt the endothelial glycocalyx and induce endothelial activation (10,27). The endotheliopathy of trauma has been predicted to occur within minutes after injury (8), and has been associated with coagulopathy (9), fibrinolysis (28), MODS (8), and increased mortality (11). In rodents and humans, endothelial glycocalyx shedding is often assessed by measuring circulating syndecan-1 levels (11,29). Although numerous assays were tested (both porcine and human), we could not reliably detect syndecan-1 in our porcine samples (data not shown). However, we were able to assess endothelial glycocalyx shedding by evaluating serum concentrations of HA (12), a glycosaminoglycan that forms an integral part of the endothelial glycocalyx (30). HA modulates a number of key endothelial functions, including barrier permeability and mechanotransduction (30). HA is shed from endothelial cells after inflammatory stimuli *in vitro* (31), is elevated after severe injury (12,32), and is associated with the development of acute traumatic coagulopathy in human patients (12). Furthermore, shed HA fragments are known to exert a wide range of biological effects, such as acting as DAMPs (33) and disrupting the endothelial barrier (34).

We demonstrate that resuscitation with blood products has a protective effect on the endothelial glycocalyx, consistent with findings from other preclinical studies. Numerous studies in cultured endothelial cells and in rodent trauma models have shown that FFP reduces glycocalyx shedding and improves endothelial barrier function (29,35,36), while transfusion of FFP reduces syndecan-1 levels in critically injured patients (37). It is currently unknown exactly what component(s) are responsible for the endothelial protective effects of FFP; however, there is evidence that factors such as fibrinogen, sphingosine-1 phosphate, and adiponectin play a role (10). There is less evidence for the endothelial protective effects of FWB or PRBCs (either alone or in combination with FFP), although there does seem to be a benefit. For example, in a rat model of trauma and hemorrhage, FWB and PRBCs reduce glycocalyx shedding compared with resuscitation with crystalloids (36,38), although the benefit of PRBCs is lost when they are washed, suggesting residual plasma may have been the contributing factor (38). In our study, all blood product treatment groups contained plasma in some form (either as part of FWB or as FFP), and all reduced HA levels to a similar extent. Treatments containing RBCs (FWB and PRBC:FFP) did not show any additional benefit compared with animals treated with FFP alone. Although this suggests the plasma component of the blood groups is conferring the benefit, additional components within the RBCs may also exert an endothelial protective effect. For example, sphingosine-1 phosphate has been shown to limit glycocalyx degradation (39), and although also present in FFP, RBCs are the major source in blood (40). The model presented here recreates a “patient that has bled” scenario (e.g., where bleeding has been controlled, for example, by the use of a tourniquet) (24). As hypoxia has also been shown to promote glycocalyx shedding (41), it is possible that resuscitation with products containing RBCs may prove more beneficial (compared with FFP alone) in protecting the endothelium where there is ongoing hemorrhage. Further studies are required to explore this in more detail.

Our model induced a robust systemic inflammatory response, as evidenced by increased circulating concentrations of HMGB-1 and IL-6, elevations in neutrophil and monocyte number, neutrophil activation, and upregulation of genes associated with inflammation, such as MMP-8, NOS3, and HMOX1 across several tissues. There was also evidence of an immunosuppressive phenotype, shown by a pronounced lymphopenia and a reduction in CD80^+^CD86^+^ monocytes (reduced ability for antigen presentation). These findings are in keeping with several preclinical and human studies that demonstrate that trauma induces simultaneous pro- and anti-inflammatory responses minutes after the insult (15,16,18). However, in contrast to other studies in pigs (42) and humans (15), we did not observe elevations in TNFα concentrations. This may be attributable to our model of THS, as it has previously been shown in pigs that individual tissue injury or hemorrhage can increase levels of TNFα, but when combined, this effect is lost (43).

We observed a robust increase in circulating neutrophil number, with concurrent degranulation, consistent with clinical studies reporting increased neutrophil number (15,44) and activation in the hours after injury (15,18,19). Compared with the naïve control group, statistically significant elevations in MPO^+^ cells (neutrophils) were present in the mucosa of the small bowel in saline-treated animals. While we observed increases in MPO^+^ cells in the liver, lungs, and kidneys in a proportion of the animals in the saline-treated group (consistent with reports by others (29,45,46)), this did not reach statistical significance. This finding is in keeping with the small and variable ICAM-1 gene expression and variability in secondary tissue injury (manuscripts in preparation), which we observed in saline-treated animals. This variability, which we also observed in other markers (e.g., Ang-2 and HMGB-1), is consistent with the previously reported physiological responses to saline resuscitation in this model (24). This may be a part of the stochastic nature of the physiological responses to injury and resuscitation; one speculated explanation might be the cross-bred genetic background of the pigs used (compared with inbred rodent models).

We observed a significant reduction in circulating lymphocyte number throughout the resuscitation period, consistent with the human literature where lymphopenia has been observed from as early as 4 hours after injury (15–17). Failure to reverse lymphopenia is associated with the development of MODS (16) and increased mortality (17). Postinjury lymphopenia is often attributed to apoptosis (47); however, recent findings in a murine polytrauma model dispute this (48). It has also been suggested that lymphocytes may be redistributed into tissues; however, our findings demonstrating significant reductions in tissue-resident T cells compared with naïve animals, and also reported by others (48), would suggest this is unlikely. It is possible that circulating lymphocytes are being redistributed into lymph nodes and/or other tissues, which were not explored in the current study. A number of mediator(s) have been proposed to induce lymphopenia; for example, cortisol, which is elevated after injury (15), has been shown to induce lymphopenia in pigs (49). A study in rats suggested the posttrauma surge in HMGB-1 influences T-cell dynamics and dysfunction (50), which, given that we observed early changes in HMGB-1 levels, may be a contributing factor to the changes we have seen in the number of peripheral and tissue-resident T cells. Due to the key role that T cells in particular play in orchestrating the immune response, further studies exploring lymphocyte dynamics and function are required.

One of our main findings is that despite attenuating endothelial glycocalyx shedding and having improved hemodynamics, shock, and survival (24), resuscitation with blood or blood products had very little influence on the acute host response(s) to injury and hemorrhage compared with saline-treated animals. Although there is plenty of evidence in the literature showing that blood products restore the endothelial glycocalyx and reduce permeability (29,35), their ability to modulate inflammatory responses is less clear. Both PRBC and FFP can promote cytokine release from endothelial cells *in vitro*, an effect that is amplified in cells prestimulated with lipopolysaccharide (51). Furthermore, in a murine model of prolonged hypotensive resuscitation, FFP had no effect on plasma IL-6 concentrations compared with resuscitation with Hextend (45). However, we showed that FFP can reduce IL-6, and a murine study demonstrated resuscitation with FWB can attenuate serum cytokine (including IL-6) concentrations and lung injury compared with lactated Ringer’s solution (52). In addition, a number of studies have shown that FFP can limit neutrophil infiltration into the lungs (29,45) and kidney (46). The fact that we did not observe a major impact of blood products on the acute inflammatory response but did see a protective effect on the endothelial glycocalyx suggests that endotheliopathy is driven by other factors. Evidence from other studies suggests this is orchestrated by catecholamines that are released early after the onset of injury (53), but as we have previously shown that blood products reduce shock (24), they may also be protecting the endothelial glycocalyx by limiting the oxygen debt. The acute inflammatory response is initiated and propagated by DAMPs and pro-inflammatory cytokines that are released early after the onset of injury (7). It is likely that blood products are unable to modulate this process to a significant extent due to the overwhelming nature of the inflammatory response that occurs in the hours after injury. However, due to the acute nature of our study, we cannot speculate on the potential impact(s) of blood product resuscitation on the longer-term consequences of the acute inflammatory insult (such as MODS).

Interestingly, we did see differences in platelet number and function, as well as kidney EPO gene expression, in animals that were resuscitated with FWB. EPO gene expression was elevated in the kidney and liver, and in the kidney, this was attenuated in the FWB group compared with saline- and FFP-treated animals. As EPO gene expression is driven by hypoxia (54), this may suggest that the resuscitation with FWB is able to restore kidney tissue perfusion/oxygenation to a greater extent than with saline or FFP. However, this needs to be validated further in targeted experiments. As platelets have a short shelf life and require very specific and demanding storage requirements, FWB has been proposed as a way of delivering platelets far forward (55). This is supported by the results presented here, as although decreased compared with baseline levels, platelet number and function (in response to ADP) were higher in the FWB group compared with saline- and PRBC:FFP-treated animals during the resuscitation phase. Our work builds on a previous report that demonstrated platelets delivered via FWB can contribute to clot formation in an animal model of traumatic injury and hemorrhage (56). On top of the logistical benefits, the ability to deliver functional platelets may confer FWB an additional advantage over component therapy, especially if there is ongoing hemorrhage and coagulopathy.

One of the main strengths of our study is the inclusion of multiple resuscitation strategies (FFP, PRBC:FFP, FWB, and saline) in a single study, allowing direct comparisons to be drawn between different treatments. Very few preclinical studies have conducted such comparisons, especially when characterizing inflammatory responses. Our study, therefore, significantly improves our understanding in this area. For example, we have shown that animals resuscitated with FFP respond similarly to those resuscitated with PRBC:FFP and FWB for the majority of analytes measured. This may have important implications for mass casualty scenarios (either military or civilian), where the availability of blood products may be limited and/or the use of products containing RBCs needs to be prioritized. Furthermore, as dried plasma has previously been shown to have similar benefits to FFP (35), which, when coupled with the significant logistical advantages, may have important implications in the treatment of casualties in resource-constrained environments. However, as we have not tested freeze-dried plasma in the work presented here, further studies are required.

As with any experimental work, our study has limitations. Our study was powered on the primary outcome (survival) (24), and therefore may be underpowered to see statistically significant differences for some of the inflammatory markers. For example, there were some markers that showed clear trends towards a treatment effect but failed to reach statistical significance (e.g., HMGB-1; treatment effect: *P* = 0.054). This is likely to be emphasized by the variable response that is particularly evident in saline-treated animals. While this variability makes the modeling more difficult, this is likely to be more representative of the genetically diverse human population. In this study, we used FWB; however, it is well known that there are risks associated with potential autoimmune reactions and disease transmission when transfusing unscreened FWB (57). To mitigate against this, all blood products were forward and reverse matched to the recipient blood, and animals were monitored closely for any clinical signs of a transfusion reaction. No transfusion reactions were seen in this study. Although designed to recapitulate prolonged prehospital times relevant to military settings, our study still only examined the acute inflammatory response in the first 8 hours after injury. Therefore, we are unable to explore the longer-term consequences (such as MODS) of hemorrhage and tissue injury that are observed in human trauma patients (1–3). As early crystalloid use has been associated with MODS (3), it is possible that our study concluded too early to see the full effects of resuscitation with blood products. That said, pigs are recognized as a good model for studying immune responses to THS (58), and as our study is longer than a number of preclinical models (29,35,36,48), we were able to demonstrate temporal changes that may provide insight into longer-term consequences.

In conclusion, we have demonstrated that our porcine model of THS recapitulates host response(s) that are observed in humans, including endotheliopathy, inflammation, and alterations in circulating leukocyte populations. We show that FWB can increase platelet number and improve platelet function compared with component therapy, and so may provide a convenient way of delivering platelets far forward in military settings. We were also able to show that although blood and blood products can attenuate endothelial glycocalyx shedding, resuscitation with these products has little effect on the acute (<8 hours) host response(s), compared with treatment with saline.

## ACKNOWLEDGMENTS

The authors would like to thank Esther Ralph, Callie Wilson, and Dstl’s Clinical Laboratory for conducting HMGB-1, vWF antigen, and hematology analysis, respectively; and the wider Combat Casualty Care project team for their technical expertise in conducting the *in vivo* experiments. We would also like to thank Robert Gwyther for conducting the statistical analysis, and Henry Ashpitel, Paula Araujo de Assis, and the Animal Plant and Health Agency (Pathology & Animal Sciences Department) for immunohistochemistry analysis.

## Supplementary Material


