# Supplementary material for: Resuscitation with Blood Products Attenuates Endothelial Glycocalyx Shedding but not the Acute Inflammatory Response to Injury in a Military-relevant Preclinical Porcine Model of Traumatic Hemorrhagic Shock
Source: Shock. 2025 Oct 16;65(2):226–38. doi: 10.1097/SHK.0000000000002740 (PMC12863592; doi:10.1097/SHK.0000000000002740)
Supplement: Supplementary file 1 [file shk-65-226-s001.pdf]

Online Supplement

Supplementary Methods

Flow cytometry and imaging flow cytometry

In order to lyse red blood cells, 4.5 mL citrated whole blood was mixed with 45 mL BD Pharm Lyse™ solution (BD Biosciences, New Jersey, USA) and incubated for 5 minutes at room temperature with gentle agitation. Upon lysis, white blood cells were pelleted by centrifugation (300 x *g* for 5 minutes at room temperature) and washed in 4 mL flow buffer (5% FBS in PBS). Cells were pelleted by centrifugation (as before), re-suspended in their respective extracellular antibody stain (prepared in flow buffer) and incubated at room temperature in the dark for 30 minutes. For stains with extracellular markers only, cells were washed in flow buffer, pelleted and re-suspended in 0.1% paraformaldehyde. For panels that also included an intracellular marker, cells were washed in permeabilisation buffer (ThermoFisher Scientific, Waltham, USA), pelleted, re-suspended in the intracellular stain (prepared in permeabilisation buffer) and stained in the dark for 30 minutes at room temperature. Cells were then washed in flow buffer, pelleted and fixed in 0.1% paraformaldehyde. For conventional flow cytometry, at least 50,000 fixed cells were collected on a BD FACS Canto™ II (BD Biosciences, New Jersey, USA) and data analysed in FlowJo (version 10.9.0). For platelet-granulocyte interactions, 100,000 events were collected on an ImageStream®X MkII imaging flow cytometer (Luminex, Seattle, USA) and data analysed using IDEAS software (Version 6.2.64); the gating strategy is shown in **Supplementary Figure 1**.

Antibody panels

Supplementary Table 1: Antibody details for flow cytometry panels

Panel	Antibody	Clone	Fluorophore	Manufacturer	Product Code	Target
1	CD45	K252.1E4	AF647	Bio-Rad	MCA1222A647	Extracellular
	CD16	G7	PE	Bio-Rad	MCA1971PE	Extracellular
	Myeloperoxidase	MPO455-8E6	FITC	ThermoFisher	11-1299-42	Intracellular
2	CD45	K252.1E4	AF647	Bio-Rad	MCA1222A647	Extracellular
	CD3	PPT3	FITC	Abcam	ab24987	Extracellular
3	CD45	K252.1E4	AF647	Bio-Rad	MCA1222A647	Extracellular
	CD80	16-10A1	PE-CY7	ThermoFisher	25-0801-80	Extracellular
	CD86	IT2.2	BV421	BioLegend	305426	Extracellular

Table 2: Imaging flow cytometry staining panel for platelet-granulocyte interactions

Marker	Clone	Fluorophore	ISX Channel	Manufacturer	Product Code	Target
CD45	K252.1E4	AF647	11	Bio-Rad	MCA1222A647	Extracellular
CD61	JM2E5	FITC	2	Bio-Rad	MCA2263F	Extracellular
CD62p	Psel.KO.2.5	PE	3	Bio-Rad	MCA2418PE	Extracellular
Hoechst	N/A	N/A	7	ThermoFisher	62249	Nuclear dye

Platelet-granulocyte interactions

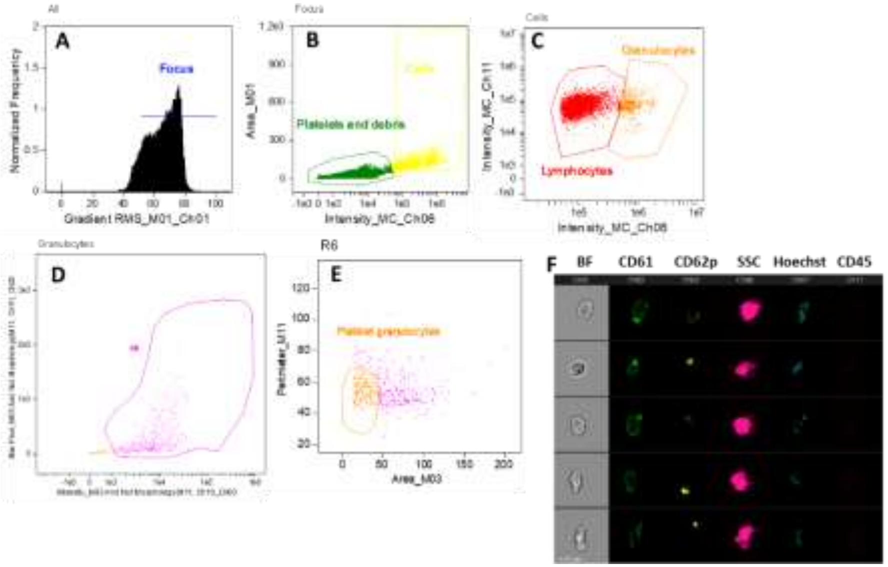

Supplementary Figure 1: platelet-granulocyte interaction gating strategy

Platelet-granulocyte interactions were assessed by imaging flow cytometry, with analysis conducted using IDEAS software (Version 6.2.64). In focus cells were gated using the Gradient_RMS feature in channel 1 (**A**). Platelets were separated from cells using side scatter intensity and area of channel 1 (**B**), and then granulocytes and lymphocytes were separated using CD45 intensity vs intensity side scatter (**C**). Granulocytes with associated CD62p were identified on granulocytes by looking at intensity of CD62p and Max_Pixel (the brightest pixel) in a surface mask (**D**). Finally to identify granulocytes with touching surface touching CD62p (platelet), area and perimeter features were used (**E**). Example images of platelet-granulocyte interactions are shown in **F**.

RT^2 Profiler PCR arrays

Initially, a 96 well custom RT^2 profiler PCR array (containing primers for 84 target genes, 5 housekeeping genes and PCR controls) was used to assess gene expression in a small number of samples across all tissues (data not shown). Preliminary data from these initial arrays was then used to create tissue-specific RT^2 profiler PCR arrays that were used for the full analysis (**Supplementary Table 3**). As well as primers for target and housekeeping genes, each array also included control wells to assess PCR performance and genomic DNA contamination.

Supplementary Table 3: Details of RT^2 PCR profiler arrays

	**Tissue**			
**Type**	**Small Bowel**	**Kidney**	**Lung**	**Liver**
Target gene	MMP-8	MMP-8	MMP-8	MMP-8
Target gene	ICAM-1	ICAM-1	ICAM-1	ICAM-1
Target gene	NOS3	NOS3	NOS3	NOS3
Target gene	SELE	HMOX-1	HMOX1	HMOX1
Target gene	IL-6	EPO	IL-1β	EPO
Target gene	IL-1β	THPO	TIMP-1	THPO
Target gene	IL-10	IL-1β	IL-10	CXCL8
Target gene	SOD2	CXCL8	TLR9	IL-6
Target gene		VEGFα		IL-1β
Target gene		SELP		TIMP-1
Target gene		TIMP-1		MMP-1
Target gene		THBD		ALOX5
Housekeeping gene	ACTG1	ACTG1	ACTG1	ACTG1
Housekeeping gene	B2M	RPL13A	RPL13A	B2M
Housekeeping gene	HPRT1	HRPT1	HRPT1	HPRT1
Control	SGDC	SGDC	SGDC	SGDC
Control	RTC	RTC	RTC	RTC
Control	PPC	PPC	PPC	PPC

SGDC: Genomic DNA control; RTC: Reverse transcription control; PPC: Positive PCR control

Immunohistochemistry

Samples fixed in 10% neutral buffered formalin were processed and embedded in paraffin wax, and 4 µM tissue sections were cut using a rotary microtome (Shandon Finesse ME; Epredia, Cheshire, UK) onto charged slides before immunolabelling for myeloperoxidase (MPO) and CD3. Slides were de-waxed, dehydrated and endogenous peroxidase activity was quenched before antigen retrieval. Slides were blocked with normal goat serum (diluted in TBST) for 20 minutes at room temperature prior to labelling with primary antibodies, or their respective isotype controls (Supplementary Table 4). Slides were washed before and after the addition of the secondary polymer and/or antibody (each: 3 x 5 minutes in TBST), and labelled cells were visualised using 3, 3'-diaminobenzidine (Sigma-Aldrich, Burlington, USA). Slides were counterstained in haematoxylin (Pioneer Research Chemicals, Colchester, UK) rinsed in tap water and dehydrated before mounting. Slides were digitally scanned (Olympus Slideview VS200-BU; Tokyo, Japan) and the number of MPO^+ and $CD3^+$ cells quantified using QuPath (version 0.4.3). MPO^+ and $CD3^+$ cells are expressed as a percentage of the total cell count.

Supplementary Table 4: Details of primary antibodies used for immunohistochemistry

Primary Antibody	Manufacturer	Product code	Antigen retrieval method	Condition	Secondary and/or polymer
Rabbit anti-myeloperoxidase	Abcam	Ab9535	pH9 TRIS-EDTA HIER	1 hour, room temperature	Rabbit Envision
*Rabbit anti-CD3	Dako	A0452	Trypsin/Chymotrypsin enzyme Digestion	Overnight, 4°C	Rabbit Envision
**Rat anti-CD3	Abcam	ab11089	pH9 TRIS-EDTA HIER	1 hour, room temperature	Rabbit anti-rat Ig, Rabbit Envision
Rabbit IgG (isotype control)	Vector Labs	I-1000	Primary antibody-dependent	Primary antibody-dependent	Rabbit Envision
Rat IgG (isotype control)	Bio-Rad	MCA1211	pH9 TRIS-EDTA HIER	1 hour, room temperature	Rabbit anti-rat Ig, Rabbit Envision

* used for the detection of T-cells in small bowel and liver samples; ** used for the detection of T-cells in kidney and lung samples

Supplementary Results

Immune cell populations

The proportion of $CD3^+$ cells were reduced after surgery ($p < 0.001$), shock ($p = 0.002$) and during the resuscitation phase ($p < 0.001$); no differences were seen between groups at any stage of the experiment (all $p > 0.05$) (**Supplementary Figure 2B)**.The number of basophils (**Supplementary Figure 2C**) was unaffected by both surgery ($p = 0.863$) and following the shock phase ($p = 0.372$), however changes were seen during the resuscitation phase ($p < 0.001$). Here basophil number rose during the early part of resuscitation, peaking at 180 minutes and declining until the end the experiment. Interestingly, although there were no differences after surgery ($p = 0.993$) or injury ($p = 0.477$), there was an effect of treatment group during the resuscitation phase ($p < 0.001$). The numbers of basophils was significantly higher in the FWB group throughout the resuscitation phase compared to all other treatment groups (all $p < 0.001$); there were no differences seen between any of the other treatment groups (all $p > 0.05$). The number of circulating eosinophils (**Supplementary Figure 2D**) were unaffected by surgery ($p = 0.769$), but were elevated following the shock phase ($p = 0.044$) and during the resuscitation phase ($p < 0.001$), where numbers increased, peaked at 180 minutes and remained stable for the rest of the experiment. There were no differences between treatment groups at any stage throughout the experiment (all $p > 0.05$).

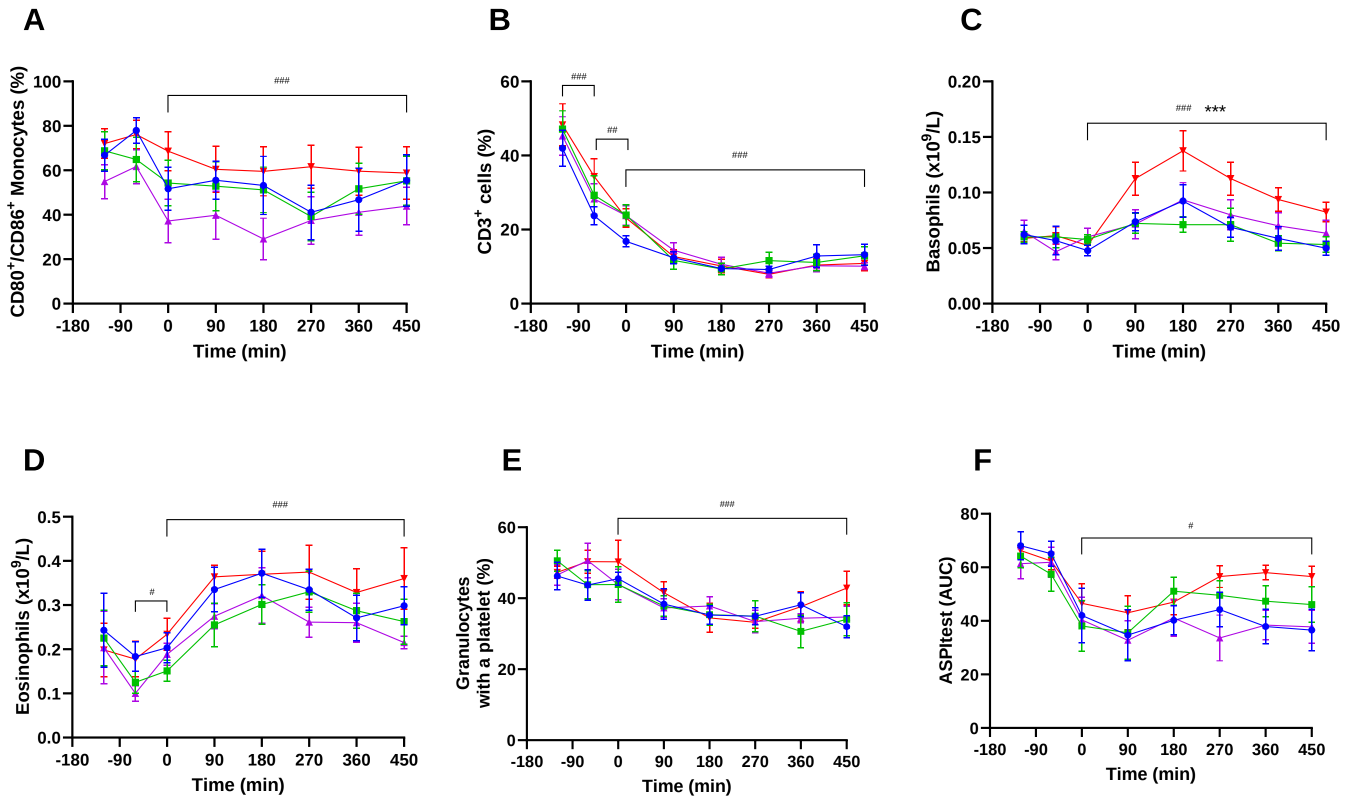

Supplementary Figure 2: Tissue injury and haemorrhagic shock alter circulating immune cell populations

The proportion of A) CD80$^+$/CD86$^+$ monocytes and B) CD3$^+$ cells was assessed by flow cytometry. The number of circulating C) basophils and D) eosinophils was assessed by haematology analysis. The proportion of E) granulocytes associated with a platelet was assessed by imaging flow cytometry and F) platelet function in response to ASPItest assessed by Multiplate. Main effect of time: # $p < 0.05$, ## $p < 0.01$, ### $p < 0.001$. Treatment effect; FWB vs saline, FWB vs PRBC:FFP and FWB vs FFP: *** $p < 0.001$. Data are presented as mean ± SEM (n = 9 per group). FFP: fresh frozen plasma; FWB: fresh whole blood; PRBC:FFP: packed red blood cells:fresh frozen plasma.

Tissue inflammation

THS (across all treatment groups and tissues) induced a significant increase in matrix metalloprotease-8 (MMP-8) (**Figures 5A**, **6A**, **7A** and **8A)** expression compared to naïve animals (all: $p < 0.001$), however there were no differences observed between treatment groups in any tissue (all: $p > 0.05$). The gene expression of intercellular adhesion molecule-1 (ICAM-1) was affected by group in the kidney (**Figure 6B**), lung (**Figure 7B**) and liver (**Figure 8B**) (all: $p < 0.05$), and there was a trend towards a difference in the small bowel (**Figure 5B**, $p = 0.069$). In the lung and liver, both saline and FFP treated animals had a significantly higher ICAM-1 gene expression than naïve animals (all: $p < 0.01$); whereas animals treated with FWB and PRBC:FFP did not (both: $p > 0.05$). There was only an effect of treatment in the kidney ($p = 0.014$), with saline-treated animals having a higher ICAM-1 expression than FFP-treated animals ($p = 0.013$); there were no other differences between treatment groups ($p > 0.05$).

With the exception of FWB ($p = 0.169$) and PRBC:FFP ($p = 0.110$) treated animals in the lung, nitric oxide synthase 3 (NOS3; **Figures 5C**, **6C**, **7C** and **8C**) expression was significantly higher in treated animals compared to naïve animals across all treatments and tissues (all: $p < 0.05$). All treatment groups significantly increased heme oxygenase 1 (HMOX1; **Figures 6D**, **7D** and **8D**) expression compared to naïve animals in the kidney, lung and liver (all: $p < 0.01$). There were no significant differences in NOS3 or HMOX1 expression between treatment groups in any of the tissues (all: $p > 0.05$).

In the kidney (**Figure 6F**) and liver (**Figure 8F**) thrombopoietin (THPO) expression in all treatment groups was significantly higher than naïve animals (all $p < 0.01$). There was a significant difference between treatment groups in the liver ($p = 0.047$); with THPO expression lower in the PRBC:FFP group compared to saline-treated animals ($p = 0.041$). There were no other differences between treatment groups (all comparisons: $p > 0.05$). In the kidney there were no significant difference between treatment groups ($p = 0.829$).

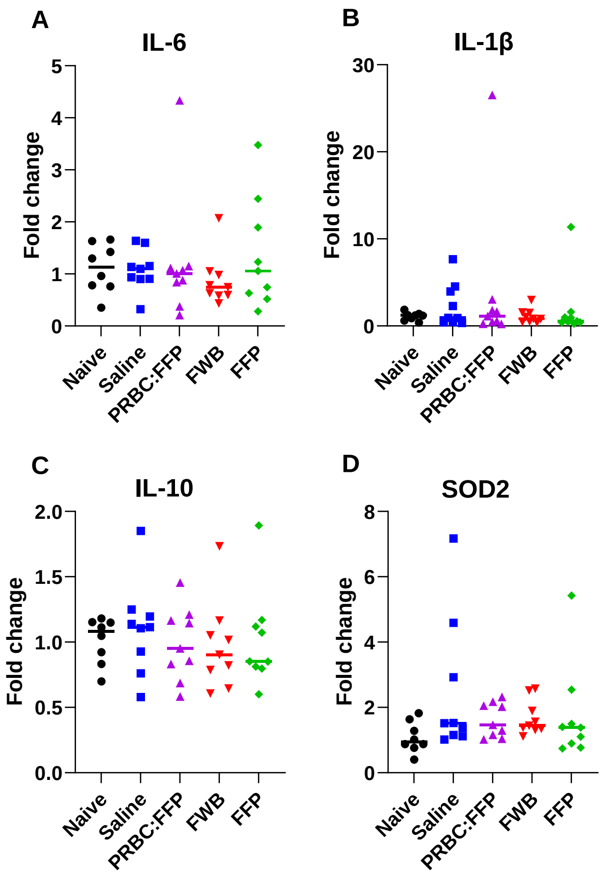

Supplementary Figure 3: Gene expression in the small bowel

Gene expression of A) IL-6, B) IL-1β, C) IL-10 and D) SOD2 in the small bowel was assessed by RT^2 profiler PCR arrays; data are presented as fold change compared to the naïve control group. Data are presented as median with individual data points (n = 9 per group). FFP: fresh frozen plasma; FWB: fresh whole blood; IL: interleukin; PRBC:FFP: packed red blood cells:fresh frozen plasma; SOD2: superoxide dismutase 2

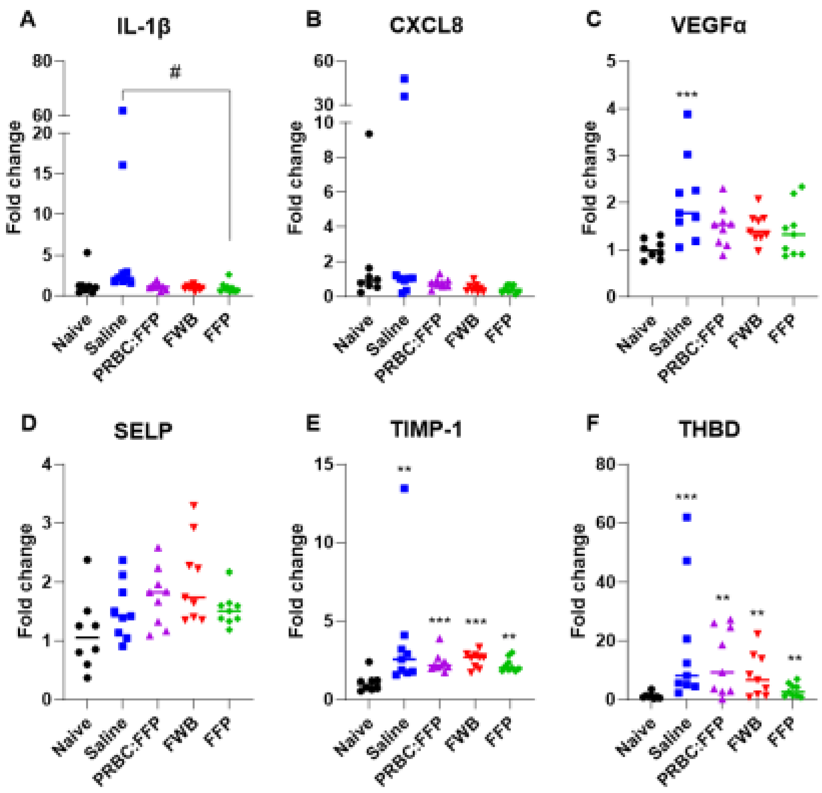

Supplementary Figure 4: Gene expression change in the kidney

Gene expression of A) IL-1β, B) CXCL8, C) VEGFα, D) SELP, E) TIMP-1 and F) THBD in the kidney was assessed by RT^2 profiler PCR arrays; data are presented as fold change compared to the naïve control group. Compared to naïve control group: * $p < 0.05$, ** $p < 0.01$, *** $p < 0.001$. Treatment comparison: # $p < 0.05$. Data are presented as median with individual data points (n = 9 per group). CXCL8: C-X-C motif ligand 8; FFP: fresh frozen plasma; FWB: fresh whole blood; IL: interleukin; PRBC:FFP: packed red blood cells:fresh frozen plasma; SELP: P-Selectin; THBD: thrombomodulin; TIMP-1: tissue inhibitor of metalloprotease-1; VEGFα: vascular endothelial growth factor α.

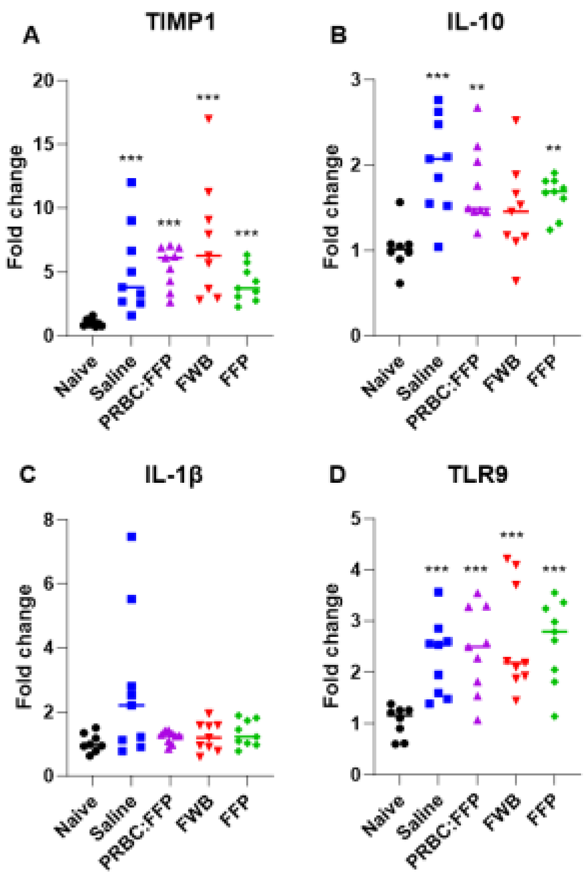

Supplementary Figure 5: Gene expression in the lung

Gene expression of A) TIMP1, B) IL-10, C) IL-1β and D) TLR9 in the lung was assessed by RT^2 profiler PCR arrays; data are presented as fold change compared to the naïve control group. Compared to naïve control group: * $p < 0.05$, ** $p < 0.01$, *** $p < 0.001$. Data are presented as median with individual data points (n = 9 per group). FFP: fresh frozen plasma; FWB: fresh whole blood; IL: interleukin; PRBC:FFP: packed red blood cells:fresh frozen plasma; TIMP1: tissue inhibitor of metalloprotease-1; TLR9: toll like receptor 9.

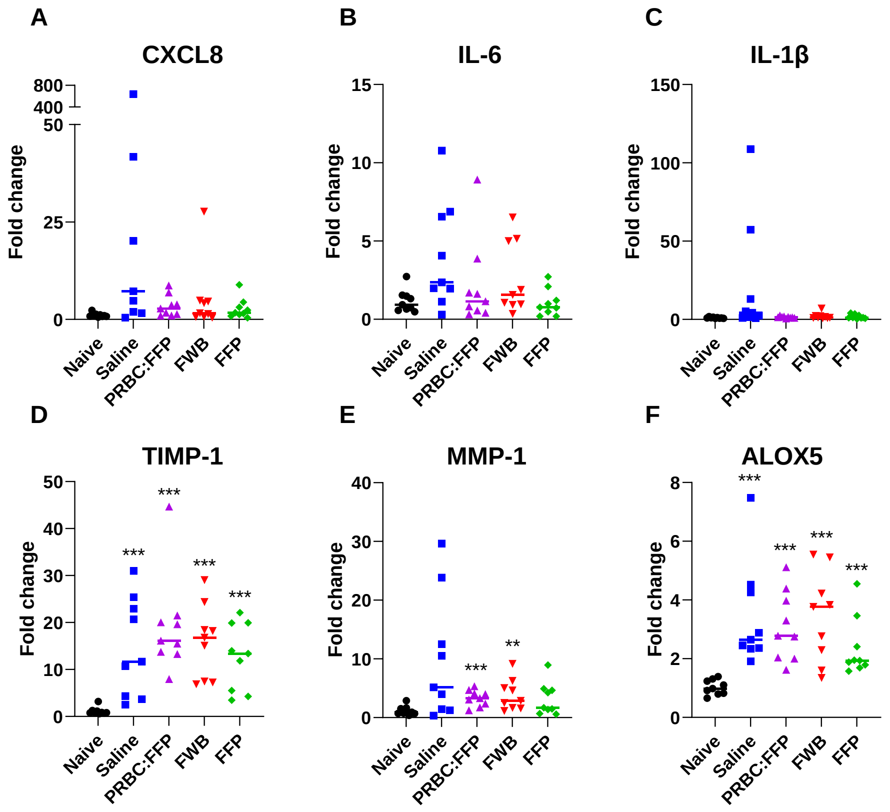

Supplementary Figure 6: Gene expression in the liver

Gene expression of A) CXCL8, B) IL-6, C) IL-1β, D), TIMP-1, E) MMP-1 and F) ALOX5 in the liver was assessed by RT^2 profiler PCR arrays; data are presented as fold change compared to the naïve control group. Compared to naïve control group: * $p < 0.05$, ** $p < 0.01$, *** $p < 0.001$. Treatment comparison: # $p < 0.05$. Data are presented as median with individual data points ($n = 9$ per group). ALOX5: arachidonate 5-lipoxygenase; CXCL8: C-X-C motif ligand 8; FFP: fresh frozen plasma; FWB: fresh whole blood; IL: interleukin; MMP1: matrix metalloprotease-1; PRBC:FFP: packed red blood cells:fresh frozen plasma; TIMP1: tissue inhibitor of metalloprotease-1.

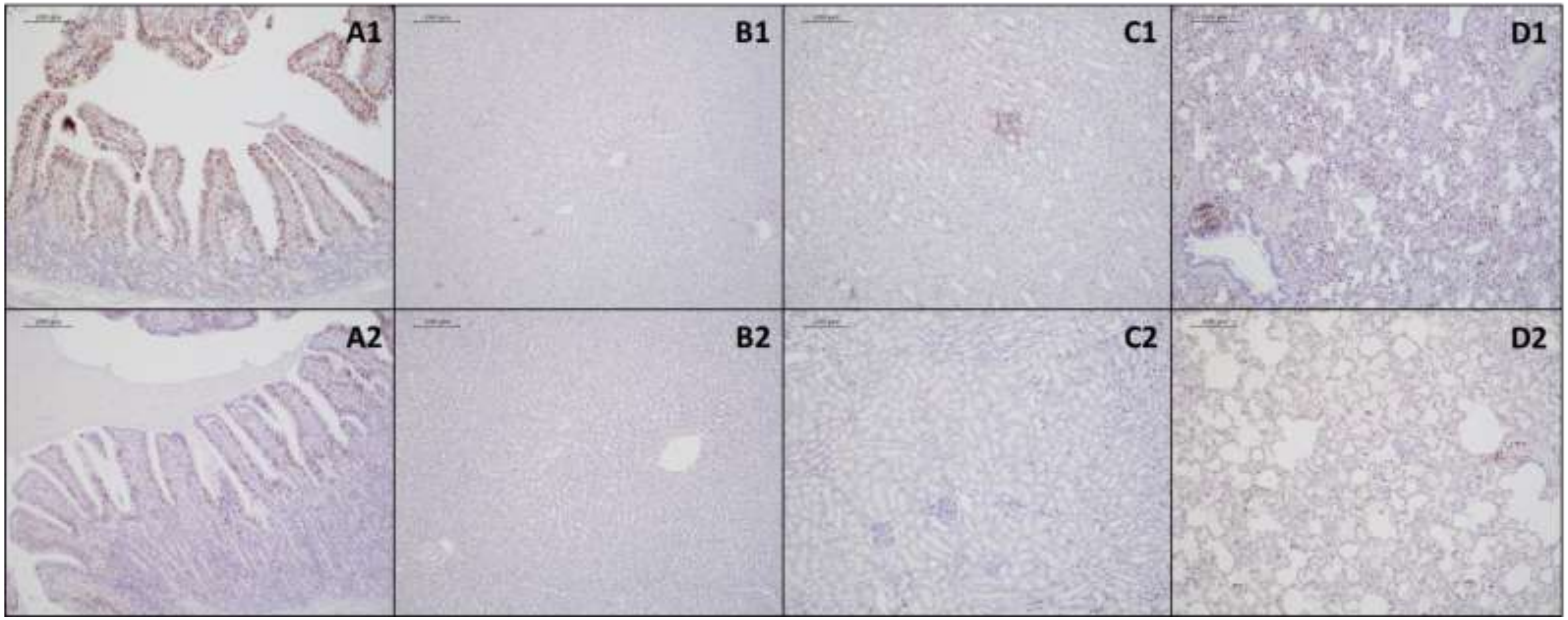

Supplementary Figure 7: images of immunohistochemical anti-CD3 antibody labelling with DAB visualisation and haematoxylin counterstain

Representative images from slides used for digital image analysis to calculate percentage of $CD3^+$ cells in each section. Scale bars in all images 200 µm. Top row: naïve control animals; bottom row: animals that received fresh whole blood (FWB) during resuscitation. A1) Small bowel mucosa from a naïve animal with numerous $CD3^+$ cells, particularly within and traversing the epithelium and lamina propria. A2) Small bowel mucosa from an animal in the FWB group, exhibiting reduced numbers of $CD3^+$ cells compared to the naïve animal. B1) Hepatic lobule from a naïve animal with occasional clusters of $CD3^+$cells within the hepatic cords. B2) Hepatic lobule from an animal in the FWB group with rare $CD3^+$cells within distended sinusoids. C1) Renal outer medulla and juxtamedullary cortex from a naïve animal with occasional clusters of $CD3^+$cells within the interstitium. C2) Renal outer medulla and juxtamedullary cortex from an animal in the FWB group, with infrequent $CD3^+$cells within the interstitium. D1) Pulmonary

parenchyma from a naïve animal with $CD3^+$ within bronchiolar associated lymphoid tissue (BALT) and within the interstitium. D2) Pulmonary parenchyma from an animal in the FWB group with lesser numbers of $CD3^+$cells within BALT and the interstitium compared to the naïve animal.
